# Thrombin Has Biphasic Effects on the Nitric Oxide-cGMP Pathway in Endothelial Cells and Contributes to Experimental Pulmonary Hypertension

**DOI:** 10.1371/journal.pone.0063504

**Published:** 2013-06-13

**Authors:** Katrin F. Nickel, Volker Laux, Rolf Heumann, Georges von Degenfeld

**Affiliations:** 1 Cardiology Research, Bayer HealthCare AG, Wuppertal, Germany; 2 Department of Molecular Medicine and Surgery, Karolinska Institutet and University Hospital, Stockholm, Sweden; 3 Biochemistry II – Molecular Neurobiochemistry, Ruhr-University Bochum, Bochum, Germany; 4 Common Mechanism Research, Bayer HealthCare AG, Wuppertal, Germany, and Institute for Research in Operative Medicine, University of Witten/Herdecke, Cologne, Germany; University of Illinois at Chicago, United States of America

## Abstract

**Background:**

A potential role for coagulation factors in pulmonary arterial hypertension has been recently described, but the mechanism of action is currently not known. Here, we investigated the interactions between thrombin and the nitric oxide-cGMP pathway in pulmonary endothelial cells and experimental pulmonary hypertension.

**Principal Findings:**

Chronic treatment with the selective thrombin inhibitor melagatran (0.9 mg/kg daily via implanted minipumps) reduced right ventricular hypertrophy in the rat monocrotaline model of experimental pulmonary hypertension. *In vitro*, thrombin was found to have biphasic effects on key regulators of the nitric oxide-cGMP pathway in endothelial cells (HUVECs). Acute thrombin stimulation led to increased expression of the cGMP-elevating factors endothelial nitric oxide synthase (eNOS) and soluble guanylate cyclase (sGC) subunits, leading to increased cGMP levels. By contrast, prolonged exposition of pulmonary endothelial cells to thrombin revealed a characteristic pattern of differential expression of the key regulators of the nitric oxide-cGMP pathway, in which specifically the factors contributing to cGMP elevation (eNOS and sGC) were reduced and the cGMP-hydrolyzing PDE5 was elevated (qPCR and Western blot). In line with the differential expression of key regulators of the nitric oxide-cGMP pathway, a reduction of cGMP by prolonged thrombin stimulation was found. The effects of prolonged thrombin exposure were confirmed in endothelial cells of pulmonary origin (HPAECs and HPMECs). Similar effects could be induced by activation of protease-activated receptor-1 (PAR-1).

**Conclusion:**

These findings suggest a link between thrombin generation and cGMP depletion in lung endothelial cells through negative regulation of the nitric oxide-cGMP pathway, possibly mediated via PAR-1, which could be of relevance in pulmonary arterial hypertension.

## Introduction

Pulmonary arterial hypertension is a life-threatening progressive disease, which is characterized by functional and structural alterations of the pulmonary arterial tree, increased pulmonary vascular resistance and subsequent right heart failure [1]. Anticoagulation with warfarin is recommended for the treatment of patients with pulmonary arterial hypertension in the American College of Chest Physicians Clinical Practice guidelines [2], [3] despite the lack of conclusive experimental or clinical evidence to support its use [4], [5]. Recently, we have shown that inhibition of the coagulation factor Xa (FXa) with the oral, direct and specific FXa inhibitor rivaroxaban reduced right ventricular (RV) hypertrophy and dysfunction in monocrotaline (MCT)-induced pulmonary hypertension [6], suggesting that activation of coagulation factors is involved in disease progression. However, the mechanism of how activated clotting factors might contribute to pulmonary arterial hypertension is not known.

Coagulation factor Xa is the critical juncture of the intrinsic and extrinsic pathways of the coagulation cascade, the first step of the final, common pathway leading to thrombin generation and fibrin formation. Besides their role in clot formation, FXa and thrombin have profound and diverse direct effects on vascular cells [7]–[12], mediated at least in part through the protease-activated receptors (PARs). In particular, both FXa and thrombin have been proposed to interact with the nitric oxide-cGMP pathway, although the reported effects are partly conflicting [13]–[15].

Nitric oxide (NO) is produced by endothelial NO-synthase (eNOS) and stimulates the soluble guanylate cyclase (sGC) to synthesize cyclic guanosine monophosphate (cGMP) which, in turn, is hydrolyzed by specific phosphodiesterases (PDEs) to the inactive 5′-GMP [16]. The nitric oxide-cGMP pathway has multiple effects on the vasculature [17], and its dysfunction leading to decreased cGMP levels has been implicated in various cardiovascular diseases including pulmonary hypertension [18], [19]. Importantly, pharmacological modulation of the nitric oxide-cGMP pathway aiming at increasing cGMP, e.g. by inhaled NO or PDE5 inhibitors, has become a clinically established and highly effective treatment paradigm [20]–[27].

In light of the recent description of the role of coagulation factor Xa in experimental pulmonary hypertension, and given the increasingly established pharmacological relevance of the nitric oxide-cGMP pathway in this disease, the goal of the present study was to investigate the interplay between both pathways in pulmonary endothelial cells and in experimental pulmonary hypertension. The findings of the present study suggest a link between the activation of coagulation factors and the nitric oxide-cGMP pathway in pulmonary endothelial cells that could be mimicked by a direct peptide activator of PAR-1, and might be of relevance in pulmonary arterial hypertension.

## Results

### Thrombin inhibition reduces RV hypertrophy in experimental pulmonary hypertension

**Figure 1:**
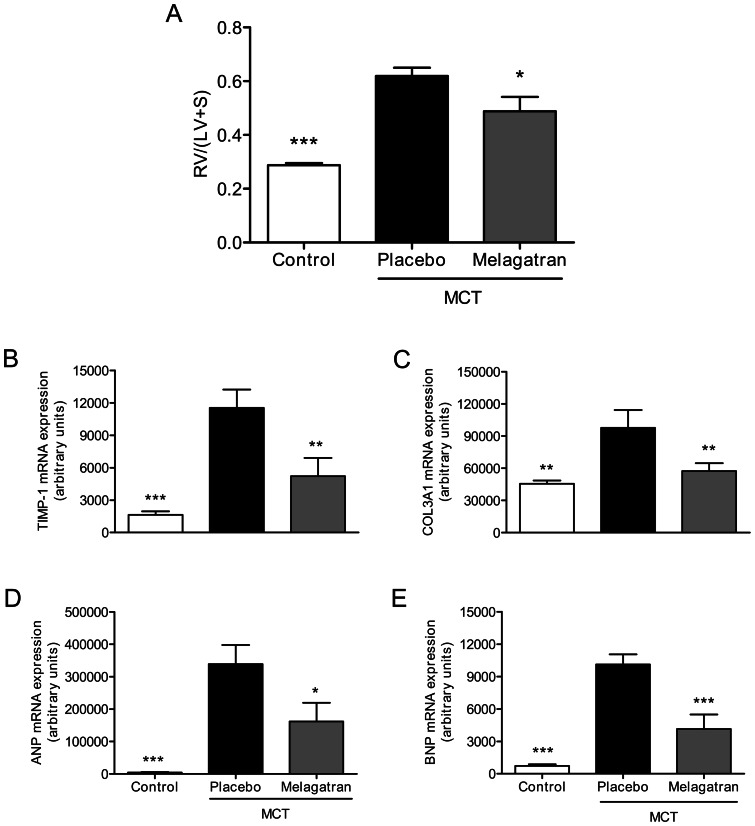
Thrombin inhibition reduces right ventricular hypertrophy in experimental pulmonary hypertension. (A) MCT-induced RV hypertrophy is significantly reduced by treatment with the selective thrombin inhibitor melagatran. Increased expression of remodeling marker genes TIMP-1 (B) and Col3A1 (C) and the pressure sensitive marker genes ANP (D) and BNP (E) in the right ventricle of rats with MCT-induced PH is reduced by melagatran. White bars: controls without MCT; black bars: MCT with placebo treatment; grey bars: MCT and melagatran treatment. Data shown as mean ± SEM (n = 8–10/group). *p<0.05, **p<0.01, **p<0.001 vs placebo.

Inhibition of coagulation factor Xa was recently shown to inhibit the development of RV hypertension and hypertrophy in the rat model of MCT-induced pulmonary hypertension [6]. Since the downstream target of FXa is prothrombin that is cleaved to active thrombin, we tested the effects of direct thrombin inhibition in the same experimental model. Rats injected with MCT were treated with the specific and direct thrombin inhibitor melagatran, administered by an osmotic minipump to provide constant plasma levels over 28 days. Here, thrombin was inhibited 54±9% at day 28 post MCT application (data not shown). One rat in each group died in the last days prior to scheduled assessment of RV hypertrophy. The MCT-injected rats treated with placebo developed significant RV hypertrophy 28 days after MCT administration: from 0.29±0.01 (controls without MCT) to 0.62±0.03 (MCT group) (p<0.001). Chronic treatment with melagatran reduced the RV hypertrophy to 0.49±0.05 (p<0.05 vs. MCT group) (Fig. 1A). In line with this, the expression of marker genes of remodeling TIMP-1 and Col3A1, and the pressure sensitive marker genes ANP and BNP were elevated in the RV of rats in the MCT-group in comparison to healthy controls, but significantly reduced under treatment with melagatran (Fig. 1B–E). These findings indicate that contribution of FXa to RV pressure increase and hypertrophy in experimental pulmonary hypertension is mediated through its downstream effector thrombin.

Cyclic GMP levels in lung homogenates were elevated from 7.7±1.0 pmol/g lung (controls without MCT) to 23.4±5.3 pmol/g lung (MCT group), confirming the previously published findings in the MCT model [28]–[30].

### Thrombin modulates the expression of key regulators of the nitric oxide-cGMP pathway in endothelial cells in a biphasic manner

**Figure 2:**
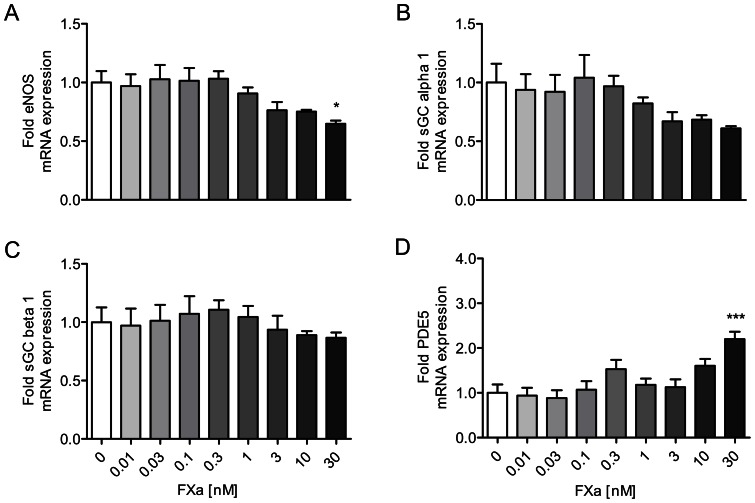
Marginal effects of FXa on key regulators of the nitric oxide-cGMP pathway in endothelial cells. Incubation of HUVECs with FXa for 6 hours reduces eNOS (A) and increases PDE5 (D) mRNA expression significantly only at the highest concentration of 30 nM. No effect on sGC alpha 1 (B) and sGC beta 1 (C) is detectable. Data shown as mean ± SEM (n = 6/group). *p<0.05, ***p<0.001 vs cells not exposed to thrombin.

First, we tested whether FXa itself directly modulates the expression levels of eNOS, sGC subunits and PDE5 in HUVECs. Although prolonged incubation with FXa reduced eNOS and increased PDE5 mRNA expression; the effects were significant only at the highest concentration of 30 nM (Fig. 2A–D). No effect on sGC alpha 1 or beta 1 levels was found. Hence, coagulation factor Xa has only marginal effects on the nitric oxide-cGMP pathway in endothelial cells.

**Figure 3:**
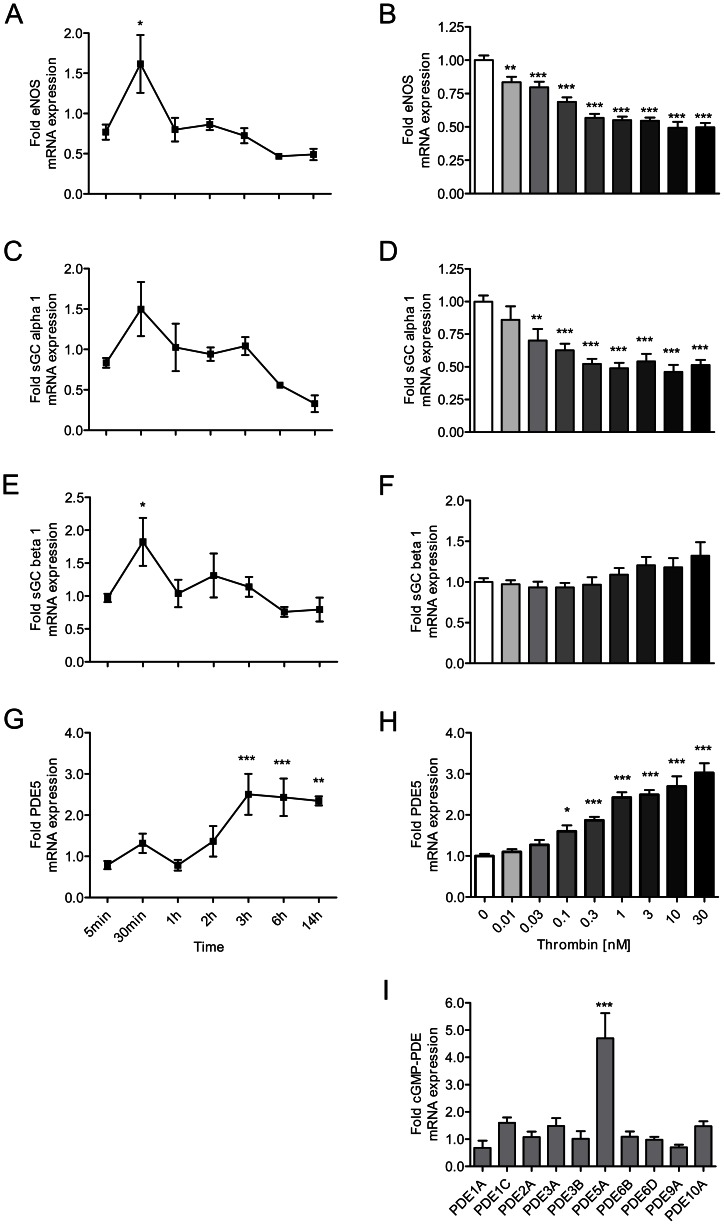
Thrombin modulates the expression of key regulators of the NO-cGMP pathway in a biphasic manner. Left panel: Short time stimulation of HUVECs with thrombin (50 nM) increases (A) eNOS and (E) sGC beta 1 expression. Right panel: By contrast, chronic stimulation decreases (B) eNOS and (D) sGC alpha 1 expression, whereas (H) PDE5 expression is increased. At late time points, all effects are concentration-dependent (B, D, H). (I) PDE5 is the only cGMP-hydrolyzing PDE to be upregulated by thrombin (30 nM). Data shown as mean ± SEM (A, C, E, G: n = 3–5/group; B, D, F, H: n = 11/group; I: n = 3/group). *p<0.05, **p<0.01, ***p<0.001 vs cells not exposed to thrombin.

Next, we stimulated endothelial cells with thrombin. Short-time stimulation of HUVECs with thrombin was found to increase the expression of eNOS and the beta 1 subunit of sGC; increased expression of the sGC subunit alpha 1 was also found, but this change did not reach the level of statistical significance. However, these effects were short-lived, and the essentially opposite pattern was found after 6 hours of incubation with thrombin: a downregulation of eNOS, confirming published findings [15] and of the alpha 1 subunit of sGC, combined with an overexpression of PDE5 (Fig. 3A–H). Interestingly, thrombin exposition selectively elevated the expression of PDE5, but not that of any other of the cGMP-hydrolyzing PDEs (Fig. 3I). Changes in expression levels were concentration-dependent, starting at thrombin concentrations as low as 10 pM (eNOS reduction), 30 pM (sGC alpha1 reduction) and 100 pM (PDE5 upregulation), respectively. Hence, exposure to thrombin revealed a striking pattern of differential expression of key regulators of the nitric oxide-cGMP pathway. Short-term stimulation led to an upregulation of factors that contribute to cGMP elevation. By contrast, prolonged incubation reduced the expression of cGMP-elevating factors, and concomitantly increased that of the cGMP-degrading PDE5.

**Figure 4:**
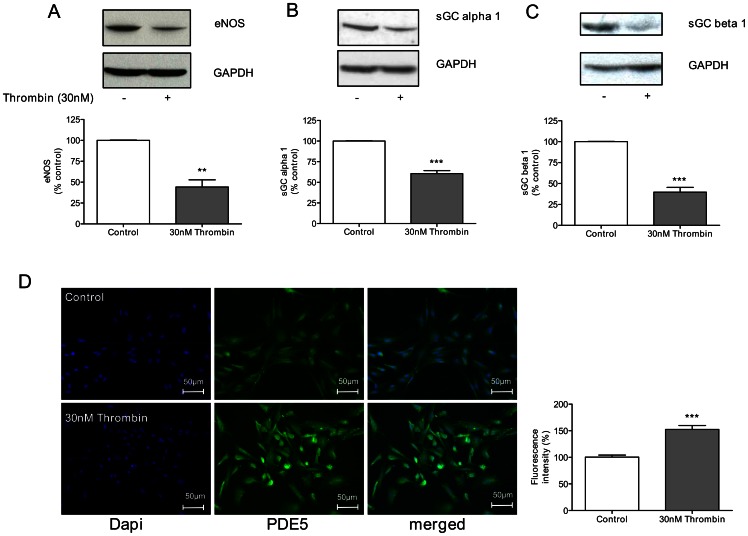
Thrombin modulates protein expression of key regulators of the nitric oxide-cGMP pathway in endothelial cells. Exposure of HUVECs to thrombin (30 nM) over 14 hours leads to a reduced expression of (A) eNOS, (B) sGC alpha 1 and (C) sGC beta 1, whereas (D) PDE5 protein expression is increased. Data shown as mean ± SEM (A, B, C: n = 5/group; D: n = 3/group). **p<0.01, ***p<0.001 vs cells not exposed to thrombin.

The protein levels of the regulators of the nitric oxide-cGMP pathway were investigated after prolonged thrombin exposure, in order to confirm the mRNA expression data. A significant decrease on eNOS (Fig. 4A) and the sGC subunits alpha 1 (Fig. 4B) and beta 1 (Fig. 4C) expression to approximately 40.1±7.8% (p<0.01), 60.6±3.1% (p<0.001) and 39.6±5.1% (p<0.001) of control level, respectively, was found by immunoblotting. Interestingly, thrombin also induced a significant decrease on sGC beta 1 (Fig. 4C) protein expression, which was not detectable at mRNA level (Fig. 3E–F). Furthermore, increased PDE5 protein expression was seen by immunofluorescence staining of cells exposed to thrombin (p<0.001) (Fig. 4D). Hence, the pattern of differential mRNA expression levels of factors of the nitric oxide-cGMP pathway was confirmed at the protein level.

### Thrombin has biphasic effects on cGMP levels in endothelial cells

**Figure 5:**
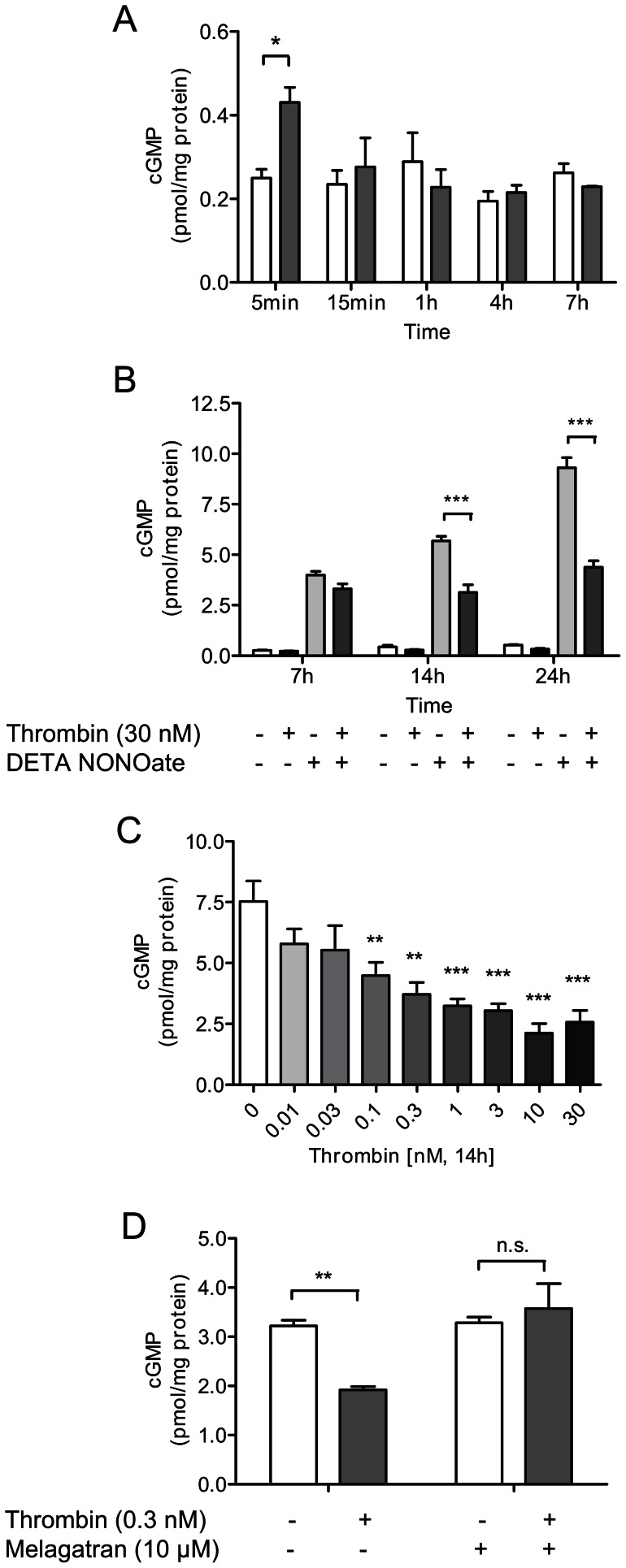
Thrombin has biphasic effects on cGMP levels in endothelial cells. (A) Thrombin (30 nM) acutely increases cGMP content in HUVECs; the effects, however, are transient (white bars: controls; grey bars: with thrombin). (B) cGMP elevation by incubation with the NO donor DETA NONOate (100 µM) is partly blunted by thrombin over 14 to 24 hours. (C) Reduction of cGMP levels in DETA NONOate-stimulated cells is concentration-dependent. (D) Thrombin-induced reduction of cGMP levels over 14 hours in DETA NONOate-stimulated cells is completely abolished by direct thrombin inhibitor melagatran (10 µM). Data shown as mean ± SEM (A, B, C: n = 3; D: n = 4/group). *p<0.05, **p<0.01, ***p<0.001 vs cells not exposed to thrombin.

Next, we investigated the functional implications of thrombin exposure. The aim was to investigate, whether the differential expression of factors of the nitric oxide-cGMP pathway seen after acute vs. prolonged thrombin exposure translated into increased and reduced cGMP levels in the expected manner. Indeed, an increase of cGMP was seen in HUVECs after 5 min of thrombin exposition (Fig. 5A), in line with the increased expression of eNOS and sGC beta 1 induced by acute thrombin exposure. The effect, however, was transient. Similarly, in cells with increased cGMP levels obtained by stimulation with IBMX, a further cGMP elevation was found by exposure to thrombin, but the thrombin effect was transient as well (Figure S1). The effects of prolonged thrombin exposure were investigated by measuring the cGMP content with thrombin exposure at 7, 14 and 24 hours in cells stimulated with DETA NONOate (Fig. 5B). NO-donation for 7, 14 and 24 hours caused an increase of cGMP at all time points, and the effect was significantly blunted by simultaneous exposure to thrombin over 14 to 24 hours (Fig. 5B). Thrombin-induced reduction of cGMP was concentration-dependent, starting at a concentration as low as 100 pM (Fig. 5C) after 14 hours of exposition. The specificity of thrombin-induced reduction of cGMP levels was verified by co-incubation with the specific thrombin inhibitor melagatran. As shown in figure 5D, the effect of thrombin was completely abolished by melagatran.

In summary, the differential regulation of key factors of the nitric oxide-cGMP pathway seen at the mRNA and protein levels was found to translate into a concordant reduction in cellular cGMP.

### Contribution of eNOS, sGC and PDE5 to thrombin-induced cGMP depletion in endothelial cells

**Figure 6:**
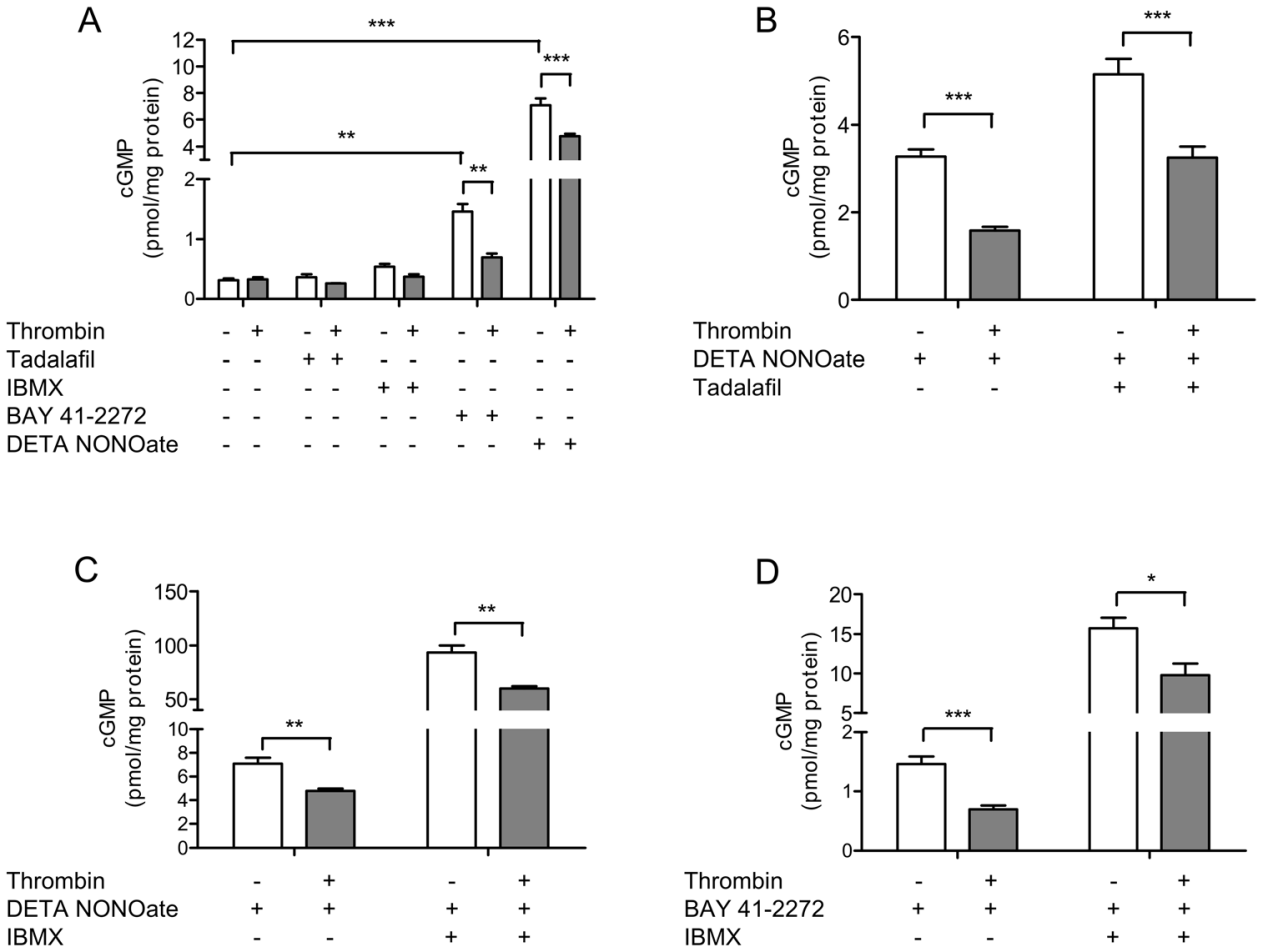
cGMP-reducing effects of thrombin in the presence of pharmacological modulators of the nitric oxide-cGMP pathway. (A) Direct sGC-stimulator BAY 41–2272 (1 µM) und NO donor DETA NONOate (100 µM) elevate cGMP, but the prolonged thrombin challenge (0.3 nM) over 14 hours still reduces cGMP (white bars: controls; grey bars: thrombin). (B) When co-stimulated with DETA NONOate, both (B) Tadalafil and (C) IBMX elevate cGMP; again, however, cGMP-decreasing effects of thrombin were still present under stimulation with these agents. (D) The combination of IBMX and BAY 41–2272 increases cGMP more strongly than either compound alone, but thrombin-induced cGMP reduction is still present. Data shown as mean ± SEM (A: n = 5/group; B: n = 12/group; C: n = 3–5/group; D: n = 5/group). *p<0.05, **p<0.01, ***p<0.001.

We sought to differentiate, whether the observed reduction of cGMP levels by prolonged thrombin stimulation were due to reduced production or increased degradation of cGMP, or both. First, we exposed HUVECs to the specific PDE5 inhibitor tadalafil and the unspecific PDE inhibitor IBMX: no significant increase in cGMP was found by either compound (Fig. 6A). By contrast, cGMP was increased by exposure to tadalafil or by IBMX when endothelial cells were co-stimulated with the NO-donor DETA NONOate (Fig. 6B, 6C). In this setting, co-incubation with thrombin significantly reduced the cGMP-elevating effect of the PDE5 inhibitor tadalafil as well as that of the unspecific PDE inhibitor IBMX. Since thrombin was found to maintain its cGMP-reducing effect even when PDE5 was inhibited, upregulation of PDE5 could be excluded as cause of thrombin-induced cGMP depletion. Next, we tested the role of factors that increase cGMP production. Incubation of endothelial cells with the direct sGC stimulator BAY 41–2272 or with the NO-donor DETA NONOate significantly increased cGMP levels (Fig. 6A). This effect was significantly blunted by thrombin. The effects were further investigated by co-stimulation with IBMX (Fig. 6C, 6D), which led to further increase of cGMP content, but, again, this effect was significantly reduced by thrombin. Since DETA NONOate and BAY 41–2272 directly stimulate the sGC, eNOS downregulation can be excluded as the driver of thrombin-induced cGMP reduction. By contrast, thrombin effects could be explained by the reduced expression of the sGC subunits. Importantly, however, cGMP levels could still be increased by drugs targeting the nitric oxide-cGMP pathway even in the presence of thrombin.

### Prolonged exposure of endothelial cells of pulmonary origin to thrombin differentially regulates key factors of the nitric oxide-cGMP pathway and causes cGMP depletion

**Figure 7:**
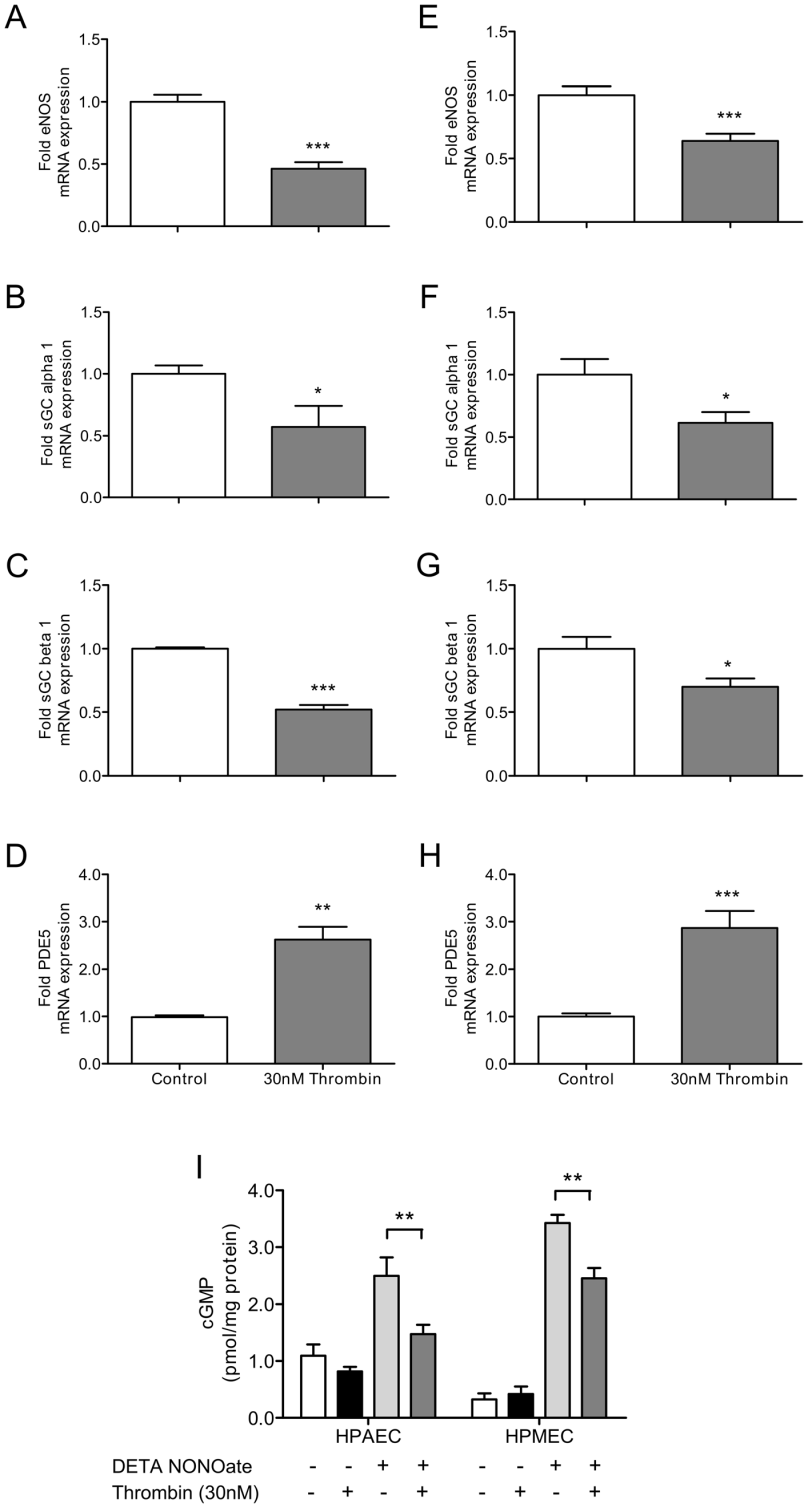
Chronic thrombin stimulation leads to nitric oxide-cGMP pathway dysfunction in pulmonary endothelial cells. Thrombin (30 nM) stimulation over 6 hours reduces the expression of (A, E) eNOS, (B, F) sGC alpha 1 and (C, G) sGC beta 1 in pulmonary artery (A–D) and pulmonary microvascular (E–H) endothelial cells, whereas (D, H) PDE5 expression is increased (White bars: control cells; grey bars: with thrombin). (I) Prolonged thrombin exposition over 14 hours leads to cGMP depletion in pulmonary endothelial cells in presence of DETA NONOate (100 µM). Data shown as mean ± SEM (A, B, C, D: n = 6/group; E, F, G, H: n = 12/group; I: n = 5–6). *p<0.05, **p<0.01 vs cells not exposed to thrombin.

Next, we tested, whether the effects seen in HUVECs could also be observed in endothelial cells of pulmonary origin: primary human pulmonary artery endothelial cells (HPAECs) and human pulmonary microvascular endothelial cells (HPMECs). Similar to the findings in HUVECs, prolonged thrombin exposure decreased eNOS (Fig. 7A, 7E) and sGC alpha 1 (Fig. 7B, 7F) expression, and increased PDE5 expression (Fig. 7D, 7H) both in HPAECs (Fig. 7A–D) and HPMECs (Fig. 7E–H). Moreover, thrombin-induced decrease of sGC beta 1 also reached the level of statistical significance, in contrast to the findings in HUVECs (Fig. 7C, 7G). Finally, prolonged thrombin exposition was found to blunt NO-induced cGMP elevation both in HPAECs and HPMECs significantly (p<0.01).

### Direct activation of PAR-1 induces changes in the nitric oxide-cGMP pathway similarly to thrombin

Thrombin is known to activate the protease-activated receptors 1, 3 and 4 [31]. To explore the mechanism by which thrombin regulates the key factors of the nitric oxide-cGMP pathway, we stimulated HUVECs with different concentration of TRAP-10, which directly and specifically activates PAR-1, and a PAR-4 activating peptide, respectively, for 6h.

**Figure 8:**
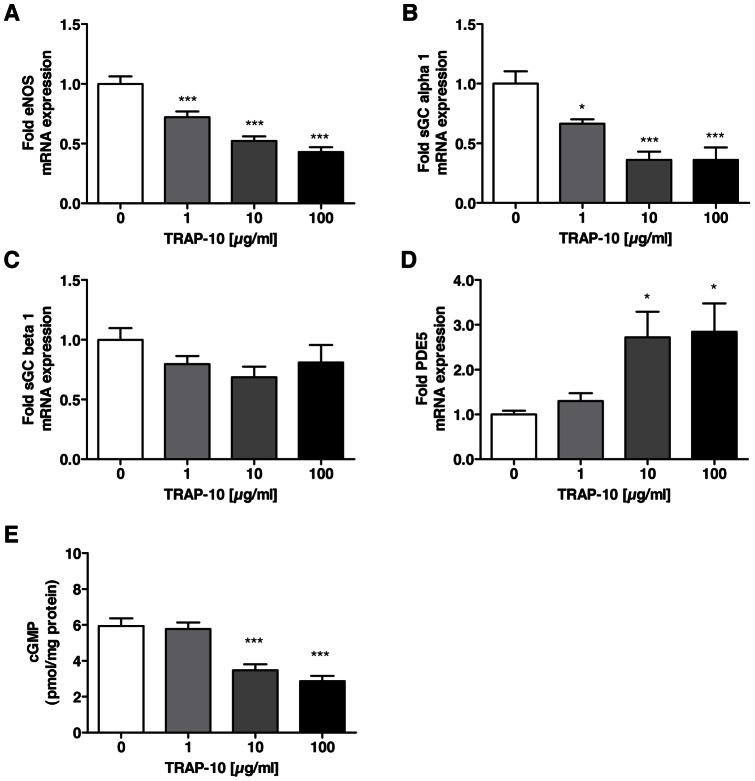
Thrombin-induced nitric oxide-cGMP pathway dysfunction is mediated via activation of protease-activated receptor-1. Exposure of HUVECs to different concentration of the PAR-1 activating peptide TRAP-1 (1 to 100 µg/ml) over 6 hours leads to reduced expression of (A) eNOS, (B) sGC alpha 1 and (C) sGC beta 1, whereas (D) PDE5 protein expression is increased. (E) Prolonged TRAP-10 exposition over 14 hours leads to cGMP depletion in HUVECs in presence of DETA NONOate (100 µM). Data shown as mean ± SEM (A, B, C, D: n = 6/group; E: n = 9/group). *p<0.05, **p<0.01, ***p<0.001 vs cells not exposed to TRAP-10.

Similar to the findings with thrombin, prolonged TRAP-10 exposure decreased eNOS (Fig. 8A) and sGC alpha 1 (Fig. 8B) expression, and increased PDE5 expression (Fig. 8D) in a concentration-dependent manner. The expression of sGC beta 1 subunit was not affected (Fig. 8C). In contrast, no effect on the nitric-oxide-cGMP pathway was detectable when HUVECs were stimulated with a PAR-4 activating peptide (data not shown). Since PAR-3 is a co-factor for the activation of the other PARs, the activation of this receptor was not analyzed.

Previously, it was shown that PAR-2 can be activated by cleaved PAR-1 and that this transactivation is recruiting PAR-2 into the thrombin response in HUVECs [32]. To analyze whether the effect of the PAR-1 activating peptide TRAP-10 on the nitric-oxide-cGMP pathway is exclusively mediated through PAR-1 activation or additionally through transactivation of PAR-2, HUVECs were also stimulated with a PAR-2 activating peptide for 6 h. The PAR-2 specific peptide did not affect the expression of eNOS, sGC alpha 1 and sGC beta 1, but increased the expression of PDE5 (data not shown).

In addition, we analyzed the effect of TRAP-10 and the PAR-2 specific peptide on cGMP level in HUVECs. In line with the results obtained after exposure to thrombin, prolonged exposure to TRAP-10 was found to blunt NO-induced cGMP elevation significantly in a concentration-dependent manner (Fig. 8E). In contrast, the exposure to the PAR-2 specific peptide did not affect the NO-induced cGMP elevation in a significant manner (data not shown).

Thus, exposure to TRAP-10 revealed a similar pattern of differential expression of key regulators of the nitric oxide-cGMP pathway, as exposure to thrombin; these data suggest that the effects of thrombin on endothelial cells in the context of pulmonary hypertension are, at least in part, mediated via PAR-1.

## Discussion

Based on the recent observation that the coagulation factor Xa is involved in RV hypertension and hypertrophy in experimental pulmonary hypertension [6], we investigated the role of its downstream effector thrombin. Even moderate chronic thrombin inhibition *in vivo* was found to also reduce RV hypertrophy in the MCT model of pulmonary hypertension, indicating that the previously shown effect of FXa is mediated through thrombin. *In vitro*, thrombin was found to have biphasic effects on the key regulators of the nitric oxide-cGMP pathway in endothelial cells. Whereas short-term exposure to thrombin resulted in an increase of cGMP, chronic exposition was found to induce the opposite effect. A pattern of differential expression of the key regulators of the nitric oxide-cGMP pathway was revealed, in which the factors contributing to cGMP elevation (eNOS and sGC) were increased by acute thrombin stimulation, whereas prolonged thrombin exposure reduced the expression of cGMP-elevating factors and increased that of the cGMP-hydrolyzing PDE5 in a remarkably specific manner. Finally, activation of PAR-1 was found to reproduce the effects seen with thrombin, indicating that thrombin-induced negative regulation of the nitric oxide-cGMP pathway in endothelial cells is, at least in part, mediated via PAR-1.

### Nitric oxide-cGMP pathway in pulmonary hypertension

The nitric oxide-cGMP pathway is known to have vasoprotective, anti-inflammatory and antioxidant effects on endothelial cells [33]–[35], and has been proposed to prevent endothelial dysfunction and intimal lesions in pulmonary arterioles of patients with pulmonary arterial hypertension [36], [37]. The clinical importance of this pathway in pulmonary arterial hypertension is well established, and drugs targeting its elements to increase cGMP levels in the pulmonary vasculature are highly therapeutically effective [22]–[27], [38], [39]. Despite these clinical successes, there is substantial controversy regarding the actual levels of expression and function of the nitric oxide-cGMP pathway in the pulmonary vasculature in animal models of pulmonary hypertension or in patients with this disease [24], [40]–[45]. In our study we observed that right ventricular hypertrophy was accompanied by the elevation of cGMP levels that confirms previous published data [28]–[30].

### Thrombin and the nitric oxide-cGMP pathway

Both FXa and thrombin have been proposed to interact with the nitric oxide-cGMP pathway in endothelial cells, although the reported effects are partly conflicting [13]–[15]. Here we sought to specifically investigate the interactions between thrombin and this pathway in the context of pulmonary arterial hypertension. Using primary endothelial cells, we found that short-term exposure to thrombin induced the increased expression of the cGMP-elevating factors eNOS and the beta 1 subunit of sGC; this effect was associated with increased cGMP as expected. However, we found these effects to be short-lived and to be reversed as early as one hour after start of thrombin exposure. By contrast, prolonged thrombin stimulation induced a striking pattern of differential expression of regulators of the nitric oxide-cGMP pathway, in which specifically the expression of factors contributing to cGMP elevation were reduced (eNOS and sGC subunits), and the expression of the cGMP-hydrolyzing PDE5 was elevated. These findings combined suggest opposite functions of acute and chronic thrombin exposition on nitric oxide-cGMP pathway in endothelial cells.

The differential expression of eNOS, sGC subunits and PDE5 seen after prolonged thrombin stimulation would be expected to work together to reduce cGMP expression which, indeed, was found to be the case in subsequent experiments. Further experiments using pharmacological tools suggested sGC-downregulation to be the main driver of cGMP lowering by thrombin, although a combined role for eNOS, sGC and PDE5 could not be ruled out. It is remarkable, however, that all three factors differentially regulated by thrombin are also specific targets for pharmacotherapy of pulmonary arterial hypertension, where the drugs act in the opposite direction of the effects seen *in vitro* for thrombin: eNOS activation (target for L-arginine), sGC stimulation (target for riociguat), and PDE5 inhibition (target for sildenafil and tadalafil) [22]–[27], [38], [39]. The relevance of the present findings for the clinical setting is unclear at present; however, it is important to note that all pharmacological compounds (NO-donors, sGC-stimulators and PDE5 inhibitors) retained their cGMP-increasing effects even in the presence of prolonged thrombin exposure.

By contrast to thrombin, chronic FXa exposition modulated only the expression of eNOS and PDE5 expression in HUVECs, and these changes were significant only at the highest concentration of 30 nM. The relevance of these changes is questionable by comparison with thrombin, which showed significant changes of eNOS, sGC subunits and PDE5 expression at concentrations as low as 10 to 100 pM. Furthermore, it is estimated that the activation of one molecule FXa results in the generation of 1000 molecules of thrombin [46] and, accordingly, the plasma concentration of FX (170 nM) is substantially lower than that of prothrombin (1.4 µM) [47]. These findings indicate that the marginal direct effect of FXa on nitric oxide-cGMP pathway in endothelial cells seen *in vitro* is most likely not physiologically relevant *in vivo*.

### A role for thrombin in pulmonary arterial hypertension?

Several authors have proposed a role for coagulation factors in the pathogenesis and progression of pulmonary arterial hypertension [4], [5], [48], [49]. Until recently, however, this concept was based solely on descriptive data from animal experiments and retrospective observations in patients with pulmonary arterial hypertension, whereas conclusive prospective interventional data were lacking.

Recently, the results from two experimental studies have reinforced the concept. A mouse overexpressing tissue factor pathway inhibitor (TFPI) was found to have reduced pulmonary hypertension when exposed to hypoxia [50]. We have recently shown that pharmacological inhibition of FXa by the direct, selective inhibitor rivaroxaban is beneficial in the MCT model of experimental pulmonary hypertension [6]. The present study is the first to show that specific inhibition of thrombin, the final enzymatic step of the coagulation cascade, is sufficient to reduce pulmonary hypertension in the MCT model.

The mode of action of thrombin in disease initiation or progression is, however, not clear. Available evidence suggests two possible mechanisms. Clot formation by thrombin-induced fibrin generation and platelet accumulation would be expected to obstruct pulmonary arteries up to total occlusion, thereby leading to resistance increase and pulmonary arterial hypertension [4], [5], [51]. In support of this hypothesis, clots are frequently found in the pulmonary vasculature of patients who have died of pulmonary arterial hypertension [52], [53]. Alternatively, a direct effect of coagulation factors on the endothelium of pulmonary arteries has been postulated. Indeed, interactions have been described between the plasma coagulation system and the endothelium, the cells that are in direct contact with plasma [7]–[9]. Specifically, FXa and thrombin have been reported to exert direct effects on endothelial cells including activation, angiogenesis, vascular leakage and inflammation [7]–[9], [21]. In line with this, thrombin has been shown to have a physiological role in modulating vascular tone by activating endothelial cells independent of its procoagulant properties [15], [54], [55].

The findings in the present study reinforce these concepts by demonstrating a dysfunction of the nitric oxide-cGMP pathway after prolonged thrombin exposition, and could indicate that direct interactions with the pulmonary endothelium might lead to a pathophysiology reminescent of “endothelial dysfunction”, ultimately leading to initiation or aggravation of pulmonary hypertension [56].

The cell-mediated effects of thrombin might be mediated through protease-activated receptors (PARs) [57]–[60]. Published evidence indicates that PAR-1 plays a role in arterial wall thickening after vascular injury [61], [62]. In addition, previous data indicate that thrombin induced a sustained contraction in normal pulmonary arteries by activating PAR-1 [63]. Recently, data were published that indicate a role for PAR-2 expressed on vascular smooth muscle cells in vascular remodeling in pulmonary hypertension [64].

We found that exposure to the PAR-1 activating peptide TRAP-10 revealed the same pattern of differential expression of key regulators of the nitric oxide-cGMP pathway as exposure to thrombin. In contrast, the exposure to a specific PAR-2 activating peptide did not influence the nitric oxide-cGMP pathway in our studies using endothelial cells. These findings indicate that the effects of thrombin on the nitroc oxide-cGNP pathway in endothelial cells might be mediated via PAR-1.

In conclusion, the findings presented here suggest a previously unrecognized link between the coagulation system and the nitric oxide-cGMP pathway, possibly mediated via PAR-1, that might be of relevance in pulmonary arterial hypertension. If confirmed by further studies, these findings could form an additional rationale for the clinical use and accepted efficacy of drugs targeting the nitric oxide-cGMP pathway, and for the additional use of anticoagulants in this indication.

## Materials and Methods

### *In Vivo* Experiments

All animal experiments were performed in accordance with the European Community guidelines for the use of experimental animals and with the German Animal Protection Act (Deutsches Tierschutzgesetz). The investigation conforms with the Guide for the Care and Use of Laboratory Animals published by the US National Institutes of Health (NIH Publication No. 85–23, revised 1996), and was approved by the Landesamt für Natur, Umwelt und Verbraucherschutz of Nordrhein-Westfalen/Germany (LANUV).

#### Monocrotaline Application and Pharmacological Treatments

Adult male Sprague-Dawley rats weighing 200 to 250 g were purchased from Charles River Laboratories (Sulzfeld, Germany). Rats were randomized to one of the treatment groups, before receiving a single subcutaneous (s.c.) injection of saline or 60 mg/kg monocrotaline (MCT) (Sigma-Aldrich Chemie GmbH, Munich, Germany) as described previously [65], [66]. The rats were randomly assigned to one of three groups (n = 10/group): (1) control animals, (2) MCT + placebo, (3) MCT + melagatran (0.9 mg/kg/d via alzet® osmotic pumps 2ML4 (Durect Corporation, Cupertino, CA, USA)) (AstraZeneca, Mölndal, Sweden). The pumps were implanted under the skin dorsally in the area of the neck under isoflorane anesthesia. All pumps were incubated in physiological saline for 24 h at 37°C before implantation. Activated pumps were then implanted one day before MCT-injection.

#### Assessment of RV Hypertrophy and Tissue Preparation

On day 28 after MCT injection, the rats were anesthetized with intraperitoneal inactin (180 mg/kg) (Sigma-Aldrich Chemie GmbH, Munich, Germany). The animals were exsanguinated via abdominal aorta and the heart was excised. The RV wall was separated from the left ventricular wall and the ventricular septum. The ratio of the right ventricle to left ventricle plus septum weight (RV/(LV+S)) was calculated as a body weight-independent index of right ventricular hypertrophy as described previously [6], [66]. The right ventricles were snap-frozen on dry ice for RNA extraction and quantitative real-time polymerase chain reaction.

#### Ecarin Fluorogenic Assay

The ecarin fluorogenic assay is a modification from the ecarin chromogenic assay (ECA) [67]. Plasma samples (20 µl), water (20 µl) and fluorogenic thrombin substrate (50 µM, 20 µl; Boc-Val-Pro-Arg-AMC; Bachem, Bubendorf, Switzerland) and Ecarin (0.4 U/ml) (Sigma-Aldrich Chemie GmbH, Munich, Germany) in Ca^2+^-Buffer (2.5 mM, 20 µl) were mixed and fluorescence was measured immediately for 20 minutes with 390 nm excitation and 465 nm emission wavelength. The thrombin inhibition was related to control plasma (0%).

### *In Vitro* Experiments

#### Cell Culture

Human umbilical vein endothelial cells (HUVECs) were purchased from Lonza (Basel, Switzerland). Human pulmonary artery endothelial cells (HPAECs) and human pulmonary microvascular endothelial cells (HPMECs) were purchased from Promocell (Heidelberg, Germany) and cultured in the medium provided from the manufacturers. For the present studies, cells between passage 2 and 5 were used. Before treatment, the cells were serum-starved for 1 hour using the adequate medium without any supplements. In all subsequent steps, cells were maintained in this serum free medium.

#### RNA Extraction, cDNA Synthesis and Quantitative Real-Time Polymerase Chain Reaction

Cells were grown in 12 multiwell plates until confluence, serum-starved for 1 hour and then treated with different concentration of either Factor Xa, thrombin (0.01 to 30 nM; Kordia, Leiden, The Netherlands), TRAP-10 (JPT Peptide Technologies, Berlin, Germany), a PAR-2 activating peptide (1 to 100 µg/ml; Bachem, Merseyside, UK) or a PAR-4 activating peptide (1 to 100 µg/ml; Bachem, Merseyside, UK) for 6 hours or with 50 nM thrombin over different time periods. Total RNA was isolated using the RNeasy Kit (Qiagen, Hilden, Germany) according to the manufacturers protocol. From snap-frozen RV samples total RNA was extracted by using TRIzol (Invitrogen, Darmstadt, Germany) [68]. Isolated RNA was reversed transcribed using Im-Prom-II Reverse Transcription System (Promega, Madison, WI, USA) and amplified using TaqMan real-time PCR as described previously [6]. Primer sequences are shown in Table S1 and detailed methodology is provided in the Materials and Methods S1.

#### Western Blot Analysis

After treatment HUVECs were harvested in ice-cold lysis buffer (Cell signaling, Danvers, MA, USA) in the presence of protease inhibitors (Complete; Roche, Basel, Switzerland). Cell lysates were subjected to Western blot analysis as described in the Materials and Methods S1 section using commercially available antibodies. eNOS (BD Transduction Laboratories, Basel, Switzerland), sGC alpha 1 (abcam, Cambridge, USA), sGC beta 1 (Cayman Chemical, Ann Arbor, Ml, USA) and GAPDH (Cell Signaling, Danvers, MA, USA). Detection was performed by the ECL method (Amersham Biosciences, Freiburg, Germany) and the protein levels were determined by densitometric analysis of the specific protein bands (GS-800 Calibrated Densitometer, Quantity One Analysis Software; Biorad, Munich, Germany).

#### Immuncytochemistry

HUVECs were plated onto gelatin (0.25%) coated glass chamber slides (Nunc, Naperville, IL, USA) and treated as above with 30 nM thrombin (Kordia, Leiden, The Netherlands). After treatment, cells were washed with PBS, fixed with 4% paraformaldehyde, permeabilized with 0.2% Triton-X 100, and stained for immunocytochemistry with a polyclonal rabbit antibody against human PDE5 (1∶1.000) (abcam, Cambridge, USA) used in conjunction with a fluorescent (Alexa 488) secondary antibody (Invitrogen, Darmstadt, Germany). Nuclei were stained with Dapi (1∶10.000; Sigma-Aldrich Chemie GmbH, Munich, Germany). The intracellular localization and expression of PDE5 at baseline and in response to thrombin was visualized by fluorescence microscopy (Axio Imager Z1; Zeiss, Göttingen, Germany) with excitation at 495 nm and emission at 519 nm for Alexa Flour 488 and excitation at 364 nm and emission at 454 nm for Dapi bound to dsDNA. In each case, fluorescence intensity was measured in levels of grey after removal of background fluorescence from four different sections by two blinded investigators using Axiovision 4 Software (Zeiss, Göttingen, Germany).

#### Measurement of cGMP

Cells were grown in 12 multiwell plates until confluence, serum-starved for 1 hour and then treated as described below.

Three different protocols were performed for treating cells before measuring of cGMP: (1) For analyzing the acute effect of thrombin, cells were exposed to 30 nM Thrombin (Kordia, Leiden, The Netherlands) over different time points in presence and absence of the unspecific PDE inhibitor IBMX (750 µM) (Sigma-Aldrich Chemie GmbH, Munich, Germany). (2) For analyzing the chronic effect of thrombin, cells were simultaneously exposed to thrombin and 100 µM of the long-acting NO-donor DETA NONOate (Cayman Chemicals, Ann Arbor, Ml, USA) (stock solution 10 mM in 0.01 mM NaOH) over different time points and different concentrations of thrombin over 14 hours, respectively. In a separate experiment, the cells were additionally incubated with the direct thrombin inhibitor melagatran, started 30 min prior thrombin and DETA NONOate exposition over 14 hours. (3) cells were exposed to thrombin over 14 hours, medium was aspirated and cells were then incubated with either the specific PDE5 inhibitor Tadalafil (10 µM) (Cialis®, Lilli Pharma, Indianapolis, IN, USA), the unspecific PDE inhibitor IBMX (750 µM) (Sigma-Aldrich Chemie GmbH, Munich, Germany), the direct sGC stimulator BAY 41–2272 (1 µM) (Bayer Schering Pharma AG, Wuppertal, Germany) or DETA NONOate for 15 minutes. Since no cGMP elevation was found with Tadalafil incubation over 15 min in combination with DETA NONOate, protocol (2) was applied for this group (2).

Following treatments, plates were placed on ice, medium was aspirated and 70% icecold EtOH were added to the wells for cGMP extraction. Plates were stored at −20°C over night and the extracts were collected. Cyclic GMP was lyophilized before the cGMP enzyme immunoassay kit (Amersham Biosciences, Freiburg, Germany) according to the manufactures instruction was performed. Cell remnants were include with 0.1 M NaOH and used for protein determination via BCA protein assay (Pierce, Rockford, USA). Values are presented at pmol/mg protein.

To measure pulmonary cGMP levels, lungs were snap-frozen on dry ice and cGMP was extracted according to the manufactures instructions of the cGMP enzyme immunoassay kit (Amersham Biosciences, Freiburg, Germany). Values are presented at pmol/g lung.

### Statistical Analysis

All data are shown as means ± SEM. Statistical comparisons between groups were performed using one-way ANOVA, followed by Student-Newman-Keuls post-hoc analysis, or an unpaired Students *t* test (two-tailed) using GraphPad Prism 5.03 Software (GraphPad Software Inc., San Diego, CA, USA). A *p* value of less than 0.05 was considered significant.

## Supporting Information

Figure S1 **Thrombin-induced cGMP elevation in IBMX-stimulated endothelial cells is transient.** Thrombin (30 nM) acutely increases cGMP content in HUVECs; the effects, however, are transient (white bars: controls; grey bars: with thrombin). Data shown as mean ± SEM (n = 3). White bars: control cells; grey bars: with thrombin. *p<0.05, ***p<0.001 vs cells not exposed to thrombin.
(TIF)

Materials and Methods S1 (DOC)

Table S1 **Primer and probe sequences for real-time PCR.**
(PPT)
