# Supplementary figures and images for: Thrombin Has Biphasic Effects on the Nitric Oxide-cGMP Pathway in Endothelial Cells and Contributes to Experimental Pulmonary Hypertension
Source: PLoS One. 2013 Jun 13;8(6):e63504. doi: 10.1371/journal.pone.0063504 (PMC3681801; doi:10.1371/journal.pone.0063504)

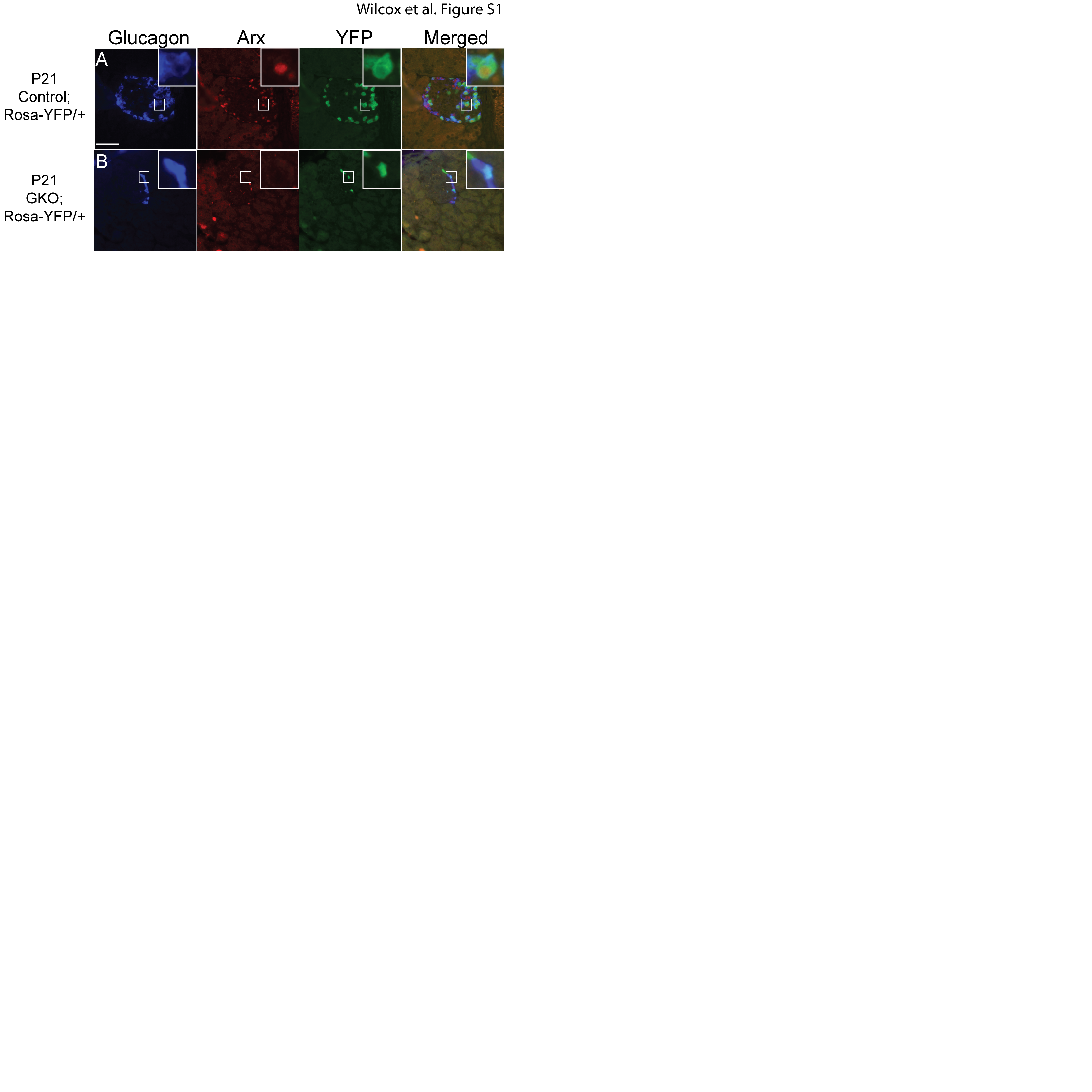

Supplement: Figure S1 — Thrombin-induced cGMP elevation in IBMX-stimulated endothelial cells is transient. Thrombin (30 nM) acutely increases cGMP content in HUVECs; the effects, however, are transient (white bars: controls; grey bars: with thrombin). Data shown as mean ± SEM (n = 3). White bars: control cells; grey bars: with thrombin. *p<0.05, ***p<0.001 vs cells not exposed to thrombin. (TIF) [file pone.0063504.s001.tif]
