# Supplementary material for: Thrombin Has Biphasic Effects on the Nitric Oxide-cGMP Pathway in Endothelial Cells and Contributes to Experimental Pulmonary Hypertension
Source: PLoS One. 2013 Jun 13;8(6):e63504. doi: 10.1371/journal.pone.0063504 (PMC3681801; doi:10.1371/journal.pone.0063504)
Supplement: Materials and Methods S1 — (DOC) [file pone.0063504.s002.doc]

**Supplemental Material and Methods:**

**cDNA Synthesis and Quantitative Real-Time Polymerase Chain Reaction**

For reverse transcription, 1 µg of total RNA (RV samples) or total volume (8 µl) (cell samples) were first digested with RNase-free DNase I (Gibco/Invitrogen, Darmstadt, Germany) for 15 minutes at room temperature and then reverse-transcribed using ImProm-II Reverse Transcription System (Promega, Madison, Wi, USA).

Real-time PCR was performed under standard cycler conditions (see TaqMan User Guide, Applied Biosytems for details) and the ABI PRISM 9600 sequence detection system. DNA sequences of PCR primers and FAM-labelled probes were designed by Primer3Plus software. Concentration of primers was 300 nM and of labelled probes 150 nM, respectively. Primer sequences are shown in Table S1. Expression was calculated using the ddCt method described by Livak and Schmittgen [69]. Ct values were corrected for ribosomal protein L32 mRNA levels to exclude different starting amounts of total RNA. The resulting expression levels for RV and cells are given in arbitrary units and fold expression relative to control, respectively.

**Western Blot Analysis**

HUVECs were grown in tissue culture dishes (75 cm^2^) until confluence, serum-starved for 1 hour and treated with 30 nM thrombin for 14 hours. Cells were washed with PBS and harvested in ice-cold lysis buffer (Cell signaling, Danvers, MA, USA) in the presence of protease inhibitors (Complete; Roche, Basel, Switzerland). Cell lysates were subjected to SDS-PAGE gel-electrophoresis and electrophoretically transferred to a nitrocellulose membrane by using the iBlot dry blotting system (Invitrogen, Darmstadt, Germany). The membranes were then blocked with 5% skim milk in TBS-Tween (0.2% Tween 20) for 1 hour and incubated either with a mouse monoclonal antibody against human eNOS (clone 3) (1:1.000) (BD Transduction Laboratories, Basel, Switzerland), a rabbit polyclonal antibody against human sGC alpha 1 (1:8.000) (abcam, Cambridge, USA), a rabbit polyclonal antibody against human sGC beta 1 (1:750) (Cayman Chemical, Ann Arbor, Ml, USA) or with the rabbit monoclonal antibody against GAPDH (1:2.000) (Cell Signaling, Danvers, MA, USA) as loading control at 4°C overnight. After being washed, the membranes were incubated with the appropriate secondary antibody conjugated to horseradish peroxidase (Cell Signaling, Danvers, MA, USA (sGC subunits); (Vectastain ABC Kit, Vectorlabs, Wiesbaden (eNOS)). Bands were visualized via chemiluminescence (Amersham Biosciences, Freiburg, Germany) and quantified by densitometric analysis of the specific protein bands (GS-800 Calibrated Densitometer, Quantity One Analysis Software; Biorad, Munich, Germany).

**References:**

[69] Livak KJ, Schmittgen TD (2001) Analysis of relative gene expression data using real-time quantitative PCR and the 2(-Delta Delta C(T)) Method Methods 25: 402-408.
