# Supplementary material for: Thrombin Has Biphasic Effects on the Nitric Oxide-cGMP Pathway in Endothelial Cells and Contributes to Experimental Pulmonary Hypertension
Source: PLoS One. 2013 Jun 13;8(6):e63504. doi: 10.1371/journal.pone.0063504 (PMC3681801; doi:10.1371/journal.pone.0063504)
Supplement: Table S1 — Primer and probe sequences for real-time PCR. (PPT) [file pone.0063504.s003.ppt]

## Slide 1
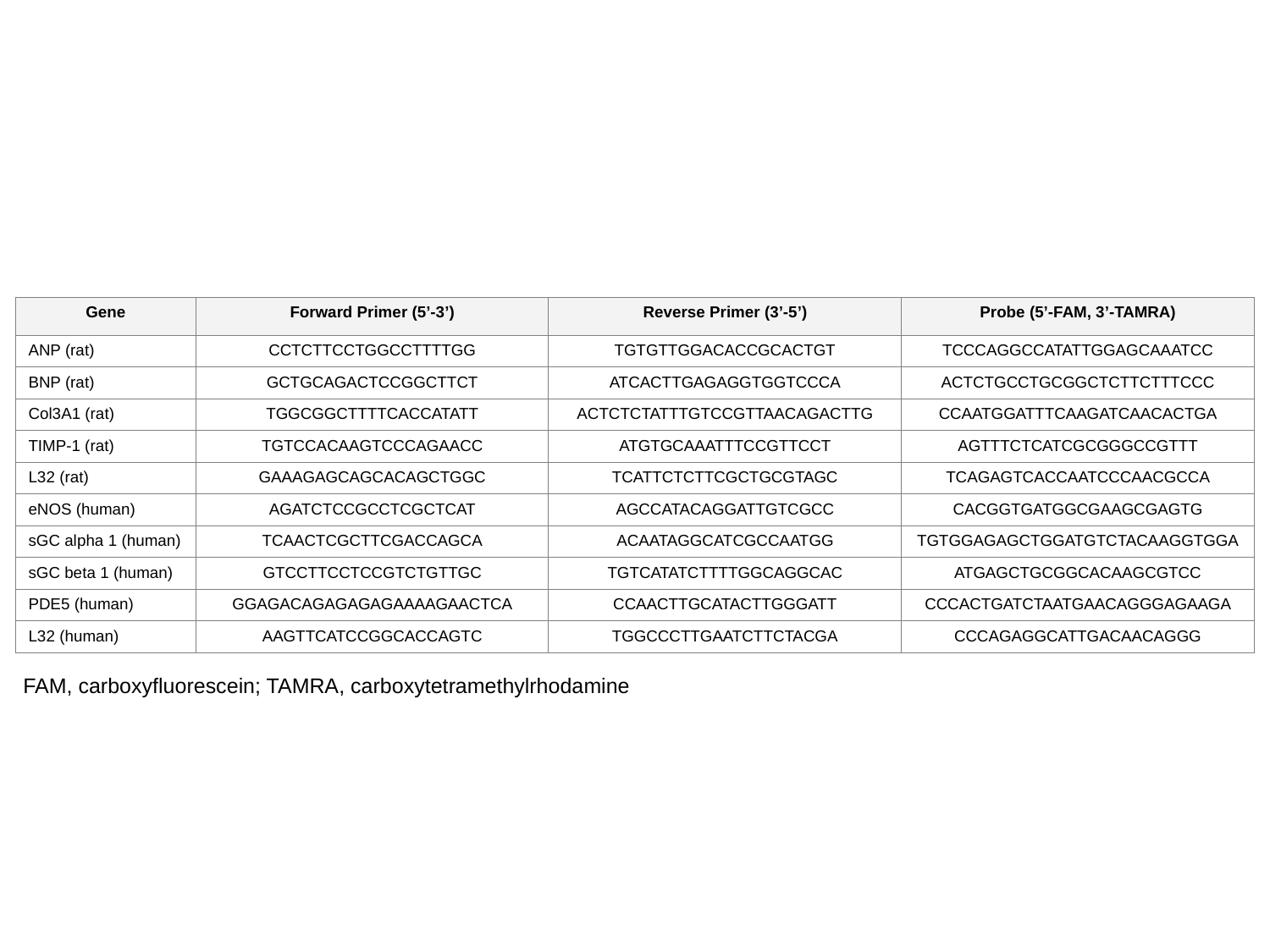

| Gene | Forward Primer (5’-3’) | Reverse Primer (3’-5’) | Probe (5’-FAM, 3’-TAMRA) |
| --- | --- | --- | --- |
| ANP (rat) | CCTCTTCCTGGCCTTTTGG | TGTGTTGGACACCGCACTGT | TCCCAGGCCATATTGGAGCAAATCC |
| BNP (rat) | GCTGCAGACTCCGGCTTCT | ATCACTTGAGAGGTGGTCCCA | ACTCTGCCTGCGGCTCTTCTTTCCC |
| Col3A1 (rat) | TGGCGGCTTTTCACCATATT | ACTCTCTATTTGTCCGTTAACAGACTTG | CCAATGGATTTCAAGATCAACACTGA |
| TIMP-1 (rat) | TGTCCACAAGTCCCAGAACC | ATGTGCAAATTTCCGTTCCT | AGTTTCTCATCGCGGGCCGTTT |
| L32 (rat) | GAAAGAGCAGCACAGCTGGC | TCATTCTCTTCGCTGCGTAGC | TCAGAGTCACCAATCCCAACGCCA |
| eNOS (human) | AGATCTCCGCCTCGCTCAT | AGCCATACAGGATTGTCGCC | CACGGTGATGGCGAAGCGAGTG |
| sGC alpha 1 (human) | TCAACTCGCTTCGACCAGCA | ACAATAGGCATCGCCAATGG | TGTGGAGAGCTGGATGTCTACAAGGTGGA |
| sGC beta 1 (human) | GTCCTTCCTCCGTCTGTTGC | TGTCATATCTTTTGGCAGGCAC | ATGAGCTGCGGCACAAGCGTCC |
| PDE5 (human) | GGAGACAGAGAGAGAAAAGAACTCA | CCAACTTGCATACTTGGGATT | CCCACTGATCTAATGAACAGGGAGAAGA |
| L32 (human) | AAGTTCATCCGGCACCAGTC | TGGCCCTTGAATCTTCTACGA | CCCAGAGGCATTGACAACAGGG |
FAM, carboxyfluorescein; TAMRA, carboxytetramethylrhodamine
